# Root and canal morphology of mandibular second molars in an Egyptian subpopulation: a cone-beam computed tomography study

**DOI:** 10.1186/s12903-023-02939-7

**Published:** 2023-04-15

**Authors:** Shehabeldin Mohamed Saber, Mohammed abou El Seoud, Shaimaa Mohamed Abu el Sadat, Nawar Naguib Nawar

**Affiliations:** 1 Department of Endodontics, Faculty of Dentistry, The British University in Egypt (BUE), El Sherouk City, Cairo Egypt; 2 Center for Innovative Dental Sciences (CIDS), Faculty of Dentistry, The British University in Egypt (BUE), Cairo, Egypt; 3 Department of Endodontics, Faculty of Dentistry, Ain Shams University, Cairo, Egypt; 4 Department of Oral and Maxillofacial Radiology, Ain Shams University, Cairo, Egypt

**Keywords:** C-shaped canal, Cone beam computed tomography, Endodontics, Mandibular second molar

## Abstract

**Background:**

This study was using Cone beam computed tomography (CBCT) to examine the anatomical variations in mandibular second molars in an Egyptian sub-population.

**Methods:**

A total of 350 CBCT images (215 females and 135 males, aged 15–65 years) of mandibular second molars were evaluated. Samples were evaluated in terms of: number of roots, number of root canals, roots’ cross section as well as prevalence and configurations of C-shaped canals. Statistical analysis was done to highlight differences between different categories and their prevalence among genders (significance level was set at *p* < 0.05). Data were presented as frequency and percentage values and were analyzed using chi square test followed by pairwise comparisons utilizing multiple Fisher’s exact tests with Bonferroni correction. The significance level was set at *p* < 0.05 within all tests.

**Results:**

Of the 350 mandibular second molars evaluated, 87.2% were non-C-shaped while 12.8% were C-shaped with no gender-based statistically significant differences (χ2 = 0.19, *p* = 0.656). Most samples had three root canals (80%) followed by two (16%), then one (3.2%), and finally four (0.8%) root canals. Among the non-C-shaped molars, presence of two roots was most common (83.4%) followed by presence of a single root (16.2%), and only one sample (0.2%) had three roots, and this had no correlation with gender (χ2 = 1.86, *p* = 0.431). In the mesial roots Type IV Vertucci was the most common configuration found (68.8%), while Type I was the most prevalent in the distal roots (91.8%). The long oval configuration was the most commonly found cross section in mesial roots while “oval” was the most prevalent in distal roots.

**Conclusion:**

Egyptian sub-population shows highly variable morphological features in mandibular second molars, hence, CBCT is highly recommended on case-to-case conditions.

## Background

Sufficient knowledge on root canal morphology is essential for recognizing and treating anatomical details within the root canal system. Root canal systems show wide variations that include the presence of extra roots, extra root canals, fins, isthmuses loops, as well as unique morphologies such as S-shaped and C-shaped canals [1, 2]. Such anatomical details can shelter microbes, thus compromising the outcome of root canal treatment [1]. Hence, it is important to comprehend root canal morphologic variations in all tooth types before treatment [2].

Mandibular second molars are amongst the teeth that exhibit a wide variation in their internal and external anatomy according to race and geographic origin [3–14]. A characteristic anatomic variant that can exist in mandibular second molars is the C-shaped canal configuration, with a wide range of reported incidence (2.7–52%) [4, 15, 16]. It was first described by Cooke and Cox [17] and was attributed to the lack of fusion of Hertwig’s root epithelial sheath of the vestibular or lingual side [17, 18]. It is characterized by a cross-sectional morphology of the pulp chamber that lacks the discrete orifices but rather resembles the letter “C”, as long as the presence of a fin that connects separate root canals [4, 19]. Moreover, C-shaped root canals have thin lingual walls, thus adding to the challenges of different root canal treatment phases [20, 21]. Mandibular second molars might also have a supernumerary root; the radix entomolaris when it exists disto-lingually, or the radix paramolaris when located mesio-buccally [22].

Different methodologies evolved throughout decades to study the morphology of root canal systems [23]. One of the technologies that helped progress this process is the Cone beam computed tomography (CBCT) which is a non-invasive technology that allows a 1:1 accurate 3-dimensional evaluation of tooth dimensions, root and canal morphology [24, 25], and it was employed to examine various tooth types both experimentally and clinically [26–29].

To date, there is scarce knowledge about the morphology of mandibular second molars in the Egyptian population. This study aimed to investigate the number of roots, root canal configurations, prevalence and morphology of the C-shaped root canal system in mandibular second molars within an Egyptian subpopulation using high- resolution CBCT.

## Methods

### Sample size calculation and study design

A power analysis was performed using G*Power version 3.1.9.7. (Heinrich-Heine-Universität, Düsseldorf, Germany) based on the results of Senan et al. [30], the predicted sample size (n) was a total of (350) cases. The study design, execution and reporting were planned to adhere to the "Preferred Reporting Items for Epidemiologic Cross-sectional Studies on Root and Root Canal Anatomy Using Cone-beam Computed Tomographic Technology" [31].

### Subjects

This study was approved and the need for consent to participate was waived by the Research Ethics Committee of The British University (El-Shorouk city, Egypt) (22–007) given the type of the retrospective study and given that the CBCT images included were anonymous. Random CBCT images for 350 patients who required a preoperative 3D radiographic assessment between January 2021 and December 2021 were collected from a private maxillofacial imaging center and included in the study. The patients were referred to the imaging center for preoperative assessment as part of their dental examination, diagnosis, and treatment planning; this included orthodontic, periodontal and surgical reasons.

### Inclusion and exclusion criteria

CBCT scans of patients who met the following criteria were included in the study: (i) Egyptian citizenship (ii) 15–65 years old (iii) presence of a fully mature and erupted mandibular second molar. Molars with root canal fillings, posts, crowns, internal or external root resorption, extensively calcified root canals, extensive coronal and root caries and periradicular radiolucency were excluded. The required sample size (*n* = 350) was reached after examination of a total of 470 CBCT scans. The excluded scans (*n* = 120 / 25% of total examined scans) did not fulfil the inclusion criteria.

### Image acquisition

High resolution CBCT scans were acquired using Planmeca Promax 3D Classic unit (Planmeca, Helsinki, Finland) with exposure settings of 90 kV, 12.5 mA, 14.966 s, Field of view (FOV) 5.0 × 5.0 cm (one side of the mandible), and voxel size: 75 μm. Images’ acquisition was done by an experienced radiologist. All data from the CBCT examinations were acquired in a digital DICOM format, imported to Planmeca Romexis dental imaging software (Planmeca, Helsinki, Finland), and viewed on an 18.5-inch HD LED monitor with a resolution of 1366 Å ~ 768.

### Calibration and measurements

Examination was performed in March 2022 as detailed in many previous studies [16, 25, 29, 30]. Three examiners (Two endodontists and an oral radiologist with more than 15 years of experience) evaluated all the scans twice. The examiners were calibrated at the beginning of the study by evaluating 15% of the scans twice with two weeks between the examinations. Any disagreement between the observers in the evaluation was resolved through discussion with a fourth observer (an endodontist with more than 10 years of experience).

### Observed morphological features

**Fig. 1:**
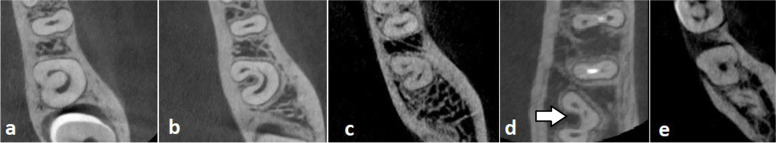
Representative axial CBCT sections showing the classification by Fan et al. [18]. 1a: class C1, a continuous C shaped canal. 1b: class C2, a mesiobuccal-distal (MB-D) ribbon-like canal and a mesiolingual (ML) canal. 1c: class C3a, a mesial (M) canal and a distal (D) canal. 1d: class C3b, a mesiobuccal (MB), a ML and a D canal. 1e: class C4, a single round or oval canal

**Fig. 2:**
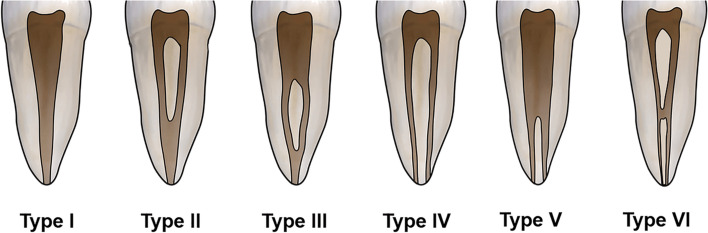
Root canal configurations detected in the study according to Vertucci’s classification (Types I to VI)

Mandibular second molars were analysed for the following: (i) number of roots, (ii) presence and prevalence of supernumerary roots; radix entomolaris or radix paramolaris, (iii) number of root canals, (iv) prevalence of C-shaped root canal system configuration in which the examined molars should have the following features: (a) fused roots, (b) a longitudinal groove on lingual or buccal surface of the root, and (c) at least one cross-section of the canal belongs to the Fan et al. [18] criteria (Fig. 1), (v) correlation of prevalence of C-shaped root canal system configuration with gender, (vi) morphology of C-shaped molars according to Fan et al. [18]; (C1): a continuous C-shaped root canal with no separation or division, (C2):a comma-shaped root canal, resulting in a non-continuous C-shaped root canal outline (mesio-buccal-distal canal and a mesio-lingual canal), (C3a): two separate root canals, (C3b): three separate root canals, (C4): only one round, oval or flat canal in the cross-section, (C5): no presence of a canal, (vii) morphology of non-C-shaped mandibular molars according to Vertucci classification (Fig. 2) [32].

### Statistical analysis

Weighted coefficient kappa (Kw) was used to measure interobserver reproducibility between observers separately for each time period and to measure intraobserver reproducibility between time periods separately for each observer. Categorical data were presented as frequency and percentage values and were analyzed using chi square test followed by pairwise comparisons utilizing multiple Fisher’s exact tests with Bonferroni correction. Confidence intervals were calculated using the Wilson score interval method [33]. The significance level was set at *p* < 0.05 within all tests. Statistical analysis was performed with R statistical analysis software version 4.1.3 for Windows.

## Results

There was an excellent intraobserver (Kw value, 0.97) and interobserver (Kw value, 0.96) reproducibility.

### Root morphology and number of roots

The initial analysis showed that 87.1% (95%CI 83.2%:90.3%) of the mandibular second molars were non-C-shaped while 12.9% (95%CI 9.7%:16.8%) were C-shaped, and this was non-significant in relation to gender (χ2 = 0.19, *p* = 0.656).

**Fig. 3:**
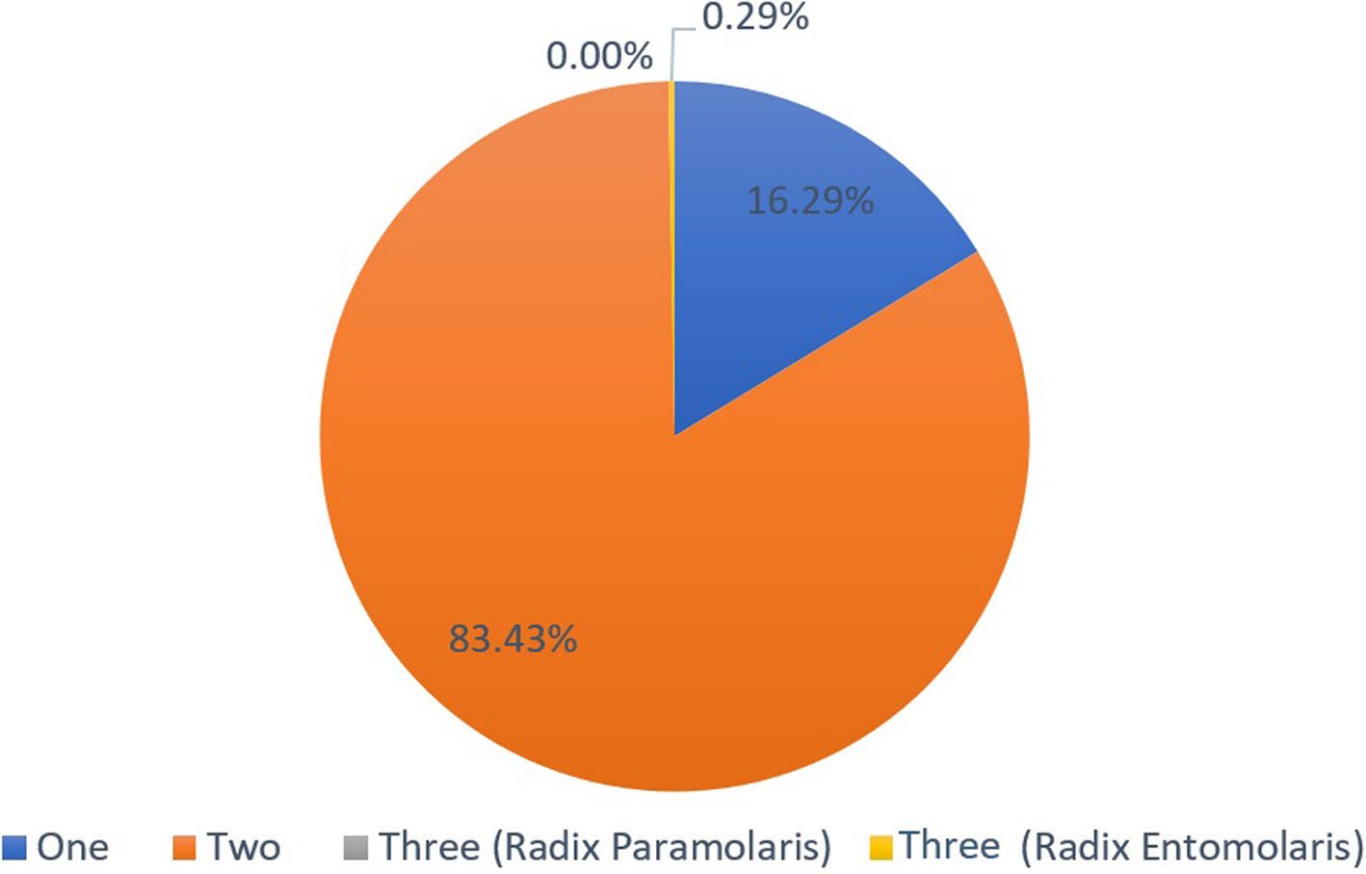
Pie chart showing number of roots and supernumerary roots

**Fig. 4:**
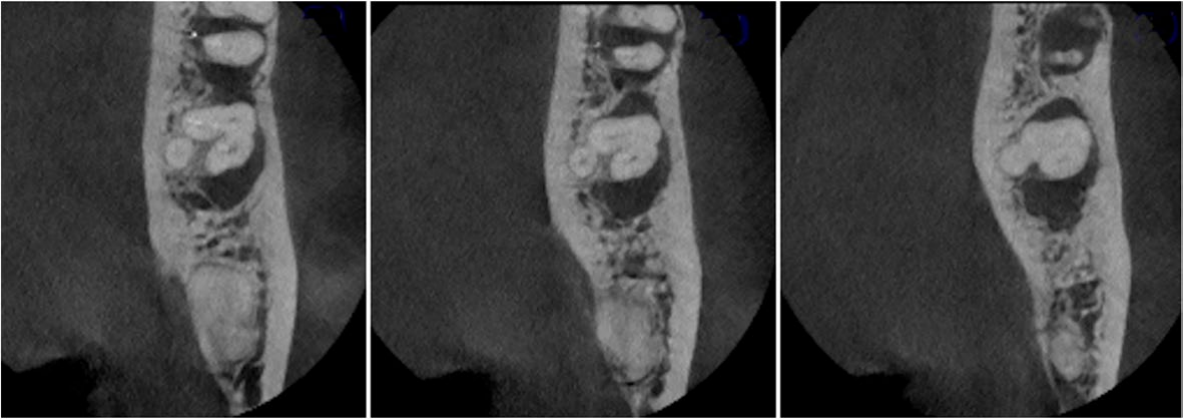
The only sample of the study showing three roots (Radix entomolaris – 0.29%)

**Fig. 5:**
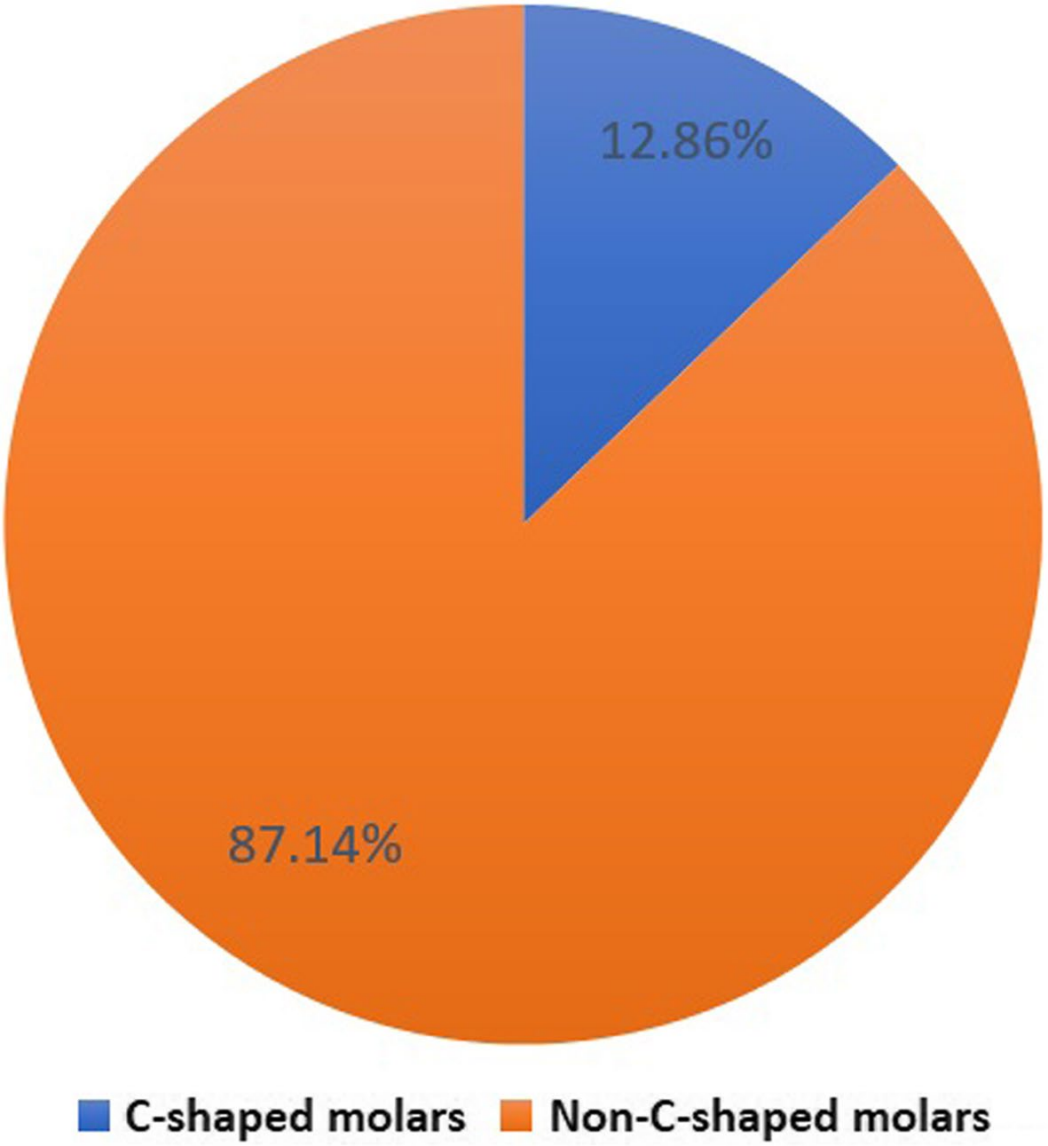
Pie chart showing the percentage of C-shaped molars

**Fig. 6:**
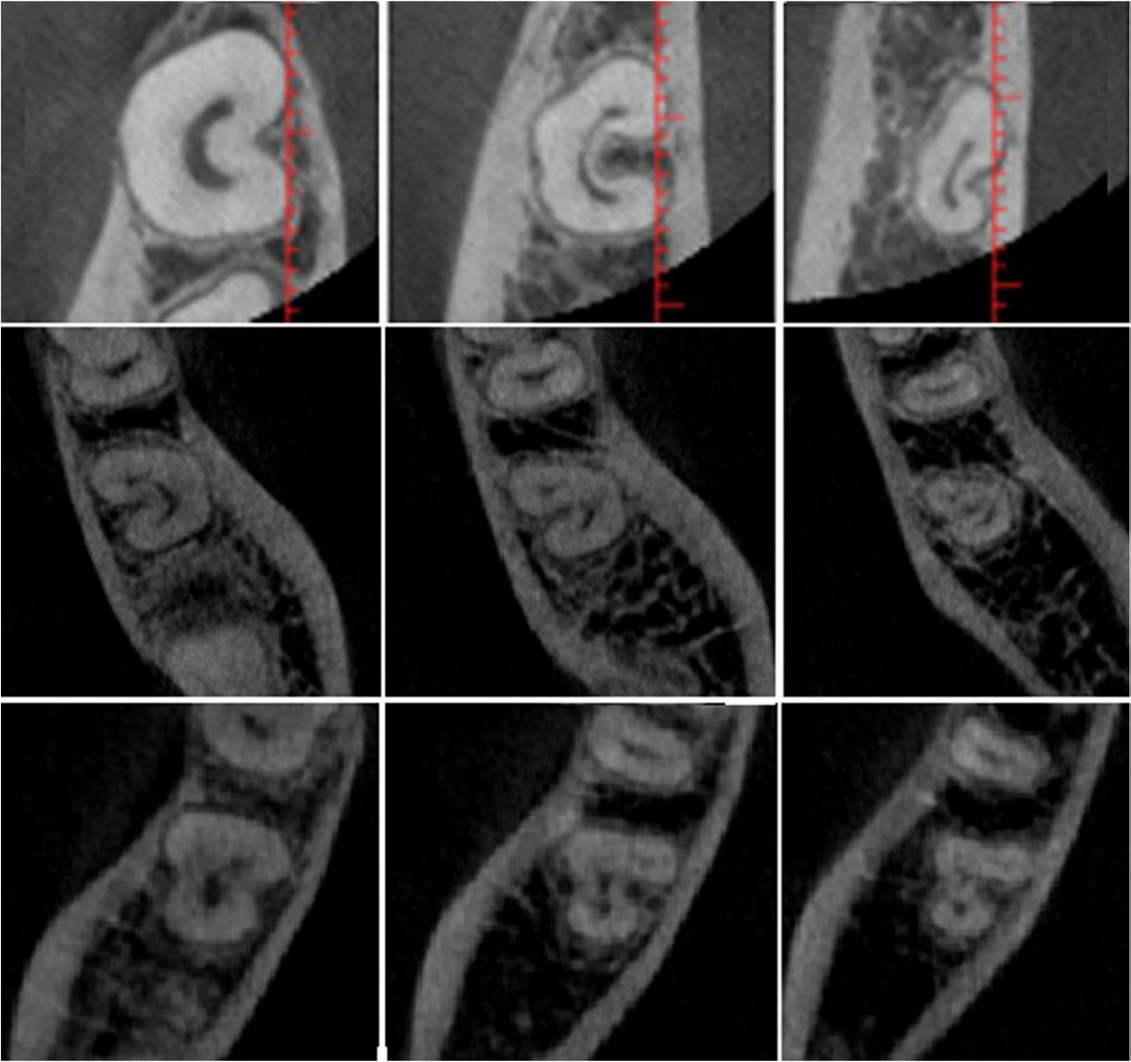
Samples of the C shaped molars included in the study

In terms of the number of roots, two roots was the most common (83.4%) (95%CI 79.2%:87.0%) followed by presence of a single root (16.3%) (95%CI 12.8%:20.5%), and only one sample (0.3%) (95%CI 0.1%:1.6%) had three roots, and this was also non-significant in relation to gender (χ2 = 1.86, *p* = 0.431) (Table 1, Figs. 3, 4, 5 and 6).

**Table 1:** Number of roots and prevalence of C-shaped anatomy in relation to gender

**Number of roots**		**Males**	**Females**	**Total**	**χ**_**2**_	***p*-value**
**One**	**n(%)**	21(6%)	36(10.2%)	57(16.3%)	**1.86**	**0.431**
**Two**	**n(%)**	109(31.1%)	183(52.2%)	292(83.4%)		
**Three** (Radix Paramolaris)	**n(%)**	0(0.0%)	0(0.0%)	0(0.0%)		
**Three** (Radix Entomolaris)	**n(%)**	1(0.2%)	0(0.0%)	1(0.3%)		
**Total**	**n(%)**	131(37.4%)	219(62.6%)	350(100.0%)		
**C-shaped**		**Males**	**Females**	**Total**	**χ**_**2**_	***p*-value**
**Yes**	**n(%)**	16(4%)	29(8.2%)	45(12.9%)	**0.19**	**0.656**
**No**	**n(%)**	119(34.0%)	186(53.1%)	305(87.1%)		

*p* = 0431 (non-significant) and *p* = 0.656 (non-significant)

### Number of roots canals

**Fig. 7:**
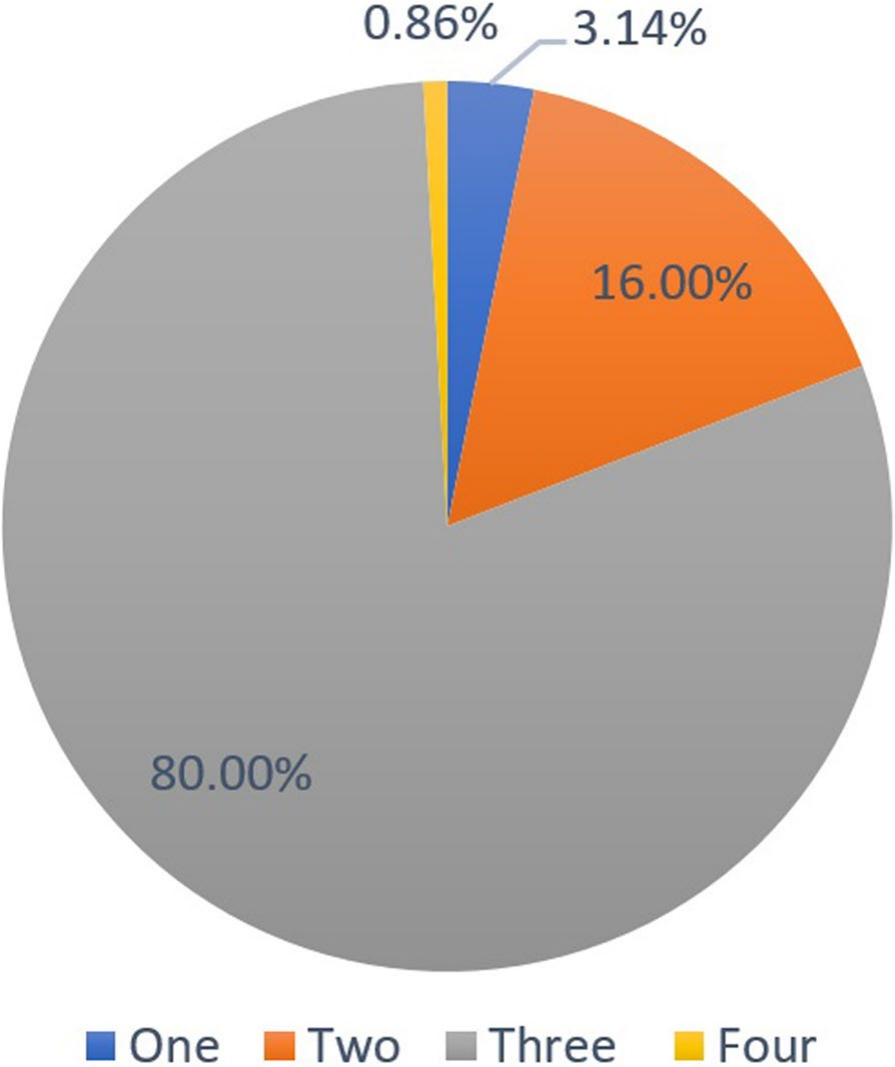
Pie chart showing the percentage of molars showing a specific number of root canals

Most samples had three (80%) (95%CI 75.5%:83.9%) followed by two (16%) (95%CI 12.5%:20.2%), then one (3.1%) (95%CI 1.8%:5.5%), and finally four (0.9%) (95%CI 0.3%:2.5%) root canals for both genders (Table 2, Fig. 7). Yet, the probability of occurrence of three root canals was significantly higher in females and the probability of one or two root canals was significantly higher in males (χ2 = 33.33, *p* < 0.001).

**Table 2:** Number of roots canals in relation to gender

		**Males**	**Females**	**Total**	**χ**_**2**_	***p*-value**
**One**	**n(%)**	8(2.2%)	3(0.9%)	11(3.1%)	**33.33**	< 0.001*
**Two**	**n(%)**	38(10.9%)	18(5.1%)	56(16%)		
**Three**	**n(%)**	87(24.9%)	193(55.1%)	280(80%)		
**Four**	**n(%)**	2(66.7%)	1(33.3%)	3(0.9%)		
**Total**	**n(%)**	135(38.6%)	215(61.4%)	350(100.0%)		

^*^significant (*p* < 0.05)

### Canal morphology of C-shaped molars

There was a significant difference between different forms of the C-shaped molars within different root sections (χ2 = 52.59, *p* < 0.001) (Table 3). Post hoc pairwise comparisons showed the C1 configuration to be the most common in the coronal one third (44.4%) while C3b was the most prevalent in both the middle and apical thirds (28.9%) and (62.2%), respectively.

**Table 3:** Prevalence of C-shaped classes according to Fan et al. (18) in different root thirds

Section		C1^A^	C2^AB^	C3a^BC^	C3b^C^	C4^C^	Total	χ_2_	***p***-value
Coronal	**n(%)**	20(44.4%)	12(26.7%)	4(8.9%)	7(15.6%)	2(4.4%)	45(100.0%)	**52.59**	< 0.001*
Middle	**n(%)**	8(17.8%)	11(24.4%)	7(15.6%)	13(28.9%)	6(13.3%)	45(100.0%)		
Apical	**n(%)**	1(2.2%)	0(0.0%)	5(11.1%)	28(62.2%)	11(24.4%)	45(100.0%)		

Groups with different superscript letters within the heading row are significantly different; *significant (*p* < 0.05)

### Root canals configuration of 2-rooted second molars according to Vertucci’s classification

There was a significant difference between different root canal configurations in different roots (χ2 = 417.72, *p* < 0.001) (Table 4). Type IV Vertucci was the most common configuration found in the mesial roots while Type I was the most prevalent one in the distal roots.

**Table 4:** Root canal configuration according to Vertucci’s classification in mandibular second molars with 2 roots (*n* = 292)

Root		I^A^	II^B^	III^B^	IV^C^	V^BC^	VI^BC^	Total	χ_2_	***p***-value
Mesial	**n(%)**	24(8.2%)	44(15.1%)	16(5.5%)	201(68.8%)	5(1.7%)	2(0.7%)	292(100.0%)	**417.72**	< 0.001*
Distal	**n(%)**	268(91.8%)	11(3.8%)	6(2.1%)	5(1.7%)	1(0.3%)	1(0.3%)	292(100.0%)		

Groups with different superscript letters within the heading row are significantly different; *significant (*p* < 0.05)

### Cross-sectional root configuration for non-C molars with 2 separate roots (*n* = 269)

For the mesial root, there was a significant difference between different root canal configurations in different sections (χ2 = 74.59, *p* < 0.001) (Table 5). The long oval configuration was the most commonly found in mesial roots while the oval cross section was the most prevalent one in distal roots; both in all three investigated levels of the root.

**Table 5:** Cross-sectional root configuration for non-C molars with 2 separate roots (*n* = 269)

**Root**	**Section**		**Bowling pin**^**A**^	**Kidney shaped**^**B**^	**Long oval**^**B**^	**Oval**^**B**^	**Ribbon**^**B**^	**Round**^**AB**^	**Total**	**χ**_**2**_	***p*-value**
**Mesial**	**Coronal**	**n(%)**	22(8.2%)	61(22.7%)	157(58.4%)	3(1.1%)	26(9.7%)	0(0.0%)	269(100.0%)	**74.59**	< 0.001*
	**Middle**	**n(%)**	47(17.5%)	66(24.5%)	156(58.0%)	0(0.0%)	0(0.0%)	0(0.0%)	269(100.0%)		
	**Apical**	**n(%)**	22(8.2%)	65(24.2%)	182(67.7%)	0(0.0%)	0(0.0%)	0(0.0%)	269(100.0%)		
**Root**	**Section**		**Bowling pin**^**ABC**^	**Kidney shaped**^**A**^	**Long oval**^**B**^	**Oval**^**C**^	**Ribbon**^**ABC**^	**Round**^**B**^	**Total**	**χ**_**2**_	***p*-value**
**Distal**	**Coronal**	**n(%)**	2(0.7%)	68(25.3%)	37(13.8%)	101(37.5%)	2(0.7%)	59(21.9%)	269(100.0%)	**219.54**	< 0.001*
	**Middle**	**n(%)**	0(0.0%)	84(31.2%)	0(0.0%)	181(67.3%)	0(0.0%)	4(1.5%)	269(100.0%)		
	**Apical**	**n(%)**	0(0.0%)	53(19.7%)	0(0.0%)	216(80.3%)	0(0.0%)	0(0.0%)	269(100.0%)		

Groups with different superscript letters within the same heading row are significantly different; *significant (*p* < 0.05)

## Discussion

The wide variation in radicular anatomy remains a major challenge in Endodontic practice [2]. These variations are not only limited to the number of root canals but also include the number of roots, root canals configuration and cross-sectional shapes [34]. These variations exist, not only between different ethnic populations, but even among individuals of the same ethnicity [35]. The Egyptian population is rich in diversity. By means of race it is considered Mediterranean, a sub-race of Caucasian, and populate the center of the Middle East-North Africa region. This study was done to address the knowledge gap related to the root and canal morphology of mandibular second molars in Egyptians.

CBCT scans were used in this investigation because of its credibility, wide acceptance and the large number of studies that employed CBCT in their methodology, thus allowing for direct comparisons [24–29]. Our inclusion criteria comprised only high-resolution, low field of view scans. This is a strength point for this study, because such imaging parameters provide better image quality and enhanced assessment of all investigated anatomic features. Another strength point is the number of experienced examiners (n = 3 + 1) who assessed the scans twice. Although micro-CT can provide further descriptive anatomical details [36], it requires more scan time with high radiation doses and is limited to ex-vivo investigations. C-shaped anatomy configuration was studied according to Fan et al. [18]. This classification was chosen over the unmodified classification by Melton et al. [37] because the former was employed in numerous studies, hence allow the possibility of direct comparisons.

Results showed that the majority of mandibular second molars in Egyptians had two roots (83.4%), A third root was prevalent in only one sample (0.29%) of the studied subpopulation. This agrees similar studies in Caucasians (1.8%-2.7%) [26, 38], but disagrees others in Eskimos, North Americans, Indians and Koreans who had higher percentages (25.3%-51.4%) of a third root [39].

Regarding the number and descriptive morphology of root canals, most samples had three (80%) followed by two (16%), then one (3.2%), and finally four (0.8%) canals. This agrees a previous study in an Egyptian subpopulation [40], as well as similar findings in Koreans [12] and Venezuelans [41], also in Turks [42] except that they found the four-canals configuration to be slightly more than the single-canal configuration.

When the internal anatomy was analysed, the mesial root showed Vertucci type IV (68.8%) configuration to be the most prevalent one in Egyptians followed by Vertucci type II (15.1%). This agrees similar findings in Turks [42] and Iranians [43] but is opposite to findings reported in Yemenis [30] and Venezuelans [41]. For the distal root, the most prevalent configuration was Vertucci type I (91.8%), which comes in accordance with most similar studies [30, 40–43].

Root cross sections were also examined and classified. The most common cross sections were long oval in the mesial root and oval in the distal root followed by the kidney shape in both. This disagrees the findings of Senan et al. [30] in Yeminis where the mesial root was mostly ribbon-shaped and the distal root was kidney-shaped. Radicular cross sections need to be studied on case-by-case basis to be able to recognize when the dentin thickness poses a threat in the dangerous zone [44].

The C-shaped molars in this study were found in 12.8% of the examined scans. This percentage is close to that reported in Belgians (10.7%) [6], Sudanese (10%) [45], Saudis (9.1%) [46], Yemenis (9%) [30], Chileans (8.9%) [6], Indians (9.7%. 8.1%) [47, 48], Brazilians (8.5%) [49] and Lebanese (9.09%) [50]. However, it was lower than results in Iranians (17.6%, 21.4%) [43, 51], Chinese (29%, 38.6%, 47.05%) [4, 52, 53], Koreans (39.8%, 44.5%) [54, 55] and Malaysians (48.7%) [56]. Nonetheless, it was higher than results in Turks (4.1%) [42]. This 12.8% prevalence of C-shaped canals contrasts another study done in an Egyptian subpopulation [40]. This contrast may be attributed to the differences in sample size, and more importantly, the imaging parameters.

The C-shaped molars were found more in female than male Egyptians with no significant difference between them. This agrees with reports from Chinese [4], Venezuelans [41], Indians [48] and Iranians [43]. However, significant gender differences was reported by Sharaan and Elrawdy in an another Egyptian subpopulation [40] and was found in Brazilians [49], Koreans [55], Saudis [46], Malaysians [56], Portuguese [13], and Turkish [16] populations with a higher incidence of C-shaped molars among females. Such contrasts emphasize the existing inter- and intra-ethnic variations and highlight the importance of CBCT as a clinical adjunct when managing individual cases.

Regarding canal configuration of C-shaped molars, C1 was the prevalent coronal configuration, while C3b was the most prevalent one in both the middle and apical thirds. This totally agrees reports in Chinese [4], Venezuelan [41] and Iranian [43] populations, partially agrees reports in Portuguese [13], Turkish [16], and Yeminis [30] populations who described different middle and apical configurations. However, disagree Alfawaz et al. [46] who found C3a to be the most prevalent coronal configuration, and C3b to be the most prevalent in middle and apical configurations in Saudis, and Kim et al. [55] who reported C2 to be the most prevalent coronal configuration in Koreans.

It is worth mentioning that this classification of Fan et al. (18) was misquoted by some studies as they used the designations C3c and C3d instead of C3a and C3b respectively [4, 30, 46, 57]. This was probably caused by the confusing labels of the demonstrative figure in Fan et al. [18] that was republished later with clearer labelling [19]. Though this remark is not exactly a finding, it is still worth mentioning as a reminder of the importance of revising primary references and providing clear illustrations.

This study adds to the scarce knowledge available about the studied population [40]. Also, the study has significant clinical relevance; Studies have shown that fused roots molars have a higher association with periapical lesions when compared to non-fused teeth [58]. Thus, the study findings that show the wide range of morphological variations justify a preoperative limited field of view CBCT for mandibular second molars once endodontic treatment is indicated. This agrees the AAE/AAOMR joint statement [59].

This study has the limitation of not investigating the ethnicity of the patients because this information was not available to collect and analyze. Also, the symmetric distribution of findings was not possible because only small field of view scans were included for the anatomic analysis. Nonetheless, even studies that showed a high probability of a patient to have C-shaped canals bilaterally still recommend individual evaluation of each mandibular second molar while performing endodontic treatment on both sides [4, 30, 40]. Finally, the impact of age, which can affect the root canal anatomy, was not included in the analysis because they were a minority in the studied population. in which about 86.6% of the patients were in the age range of 21–50 years.

In conclusion, Egyptian permanent mandibular second molars have mainly two separated mesial and distal roots with a very low prevalence of a third one. Various canals configurations were identified, with prevalence for Vertucci Type IV and Type I in the mesial and distal roots, respectively. The occurrence of C-shaped roots and canals must be considered when treating these molars, mainly in females. Also, high resolution, low field of view CBCT is a useful clinical adjunct for successful management of such teeth.

## Data Availability

The data that support the findings of this study are available from The British University—faculty of Dentistry, but restrictions apply to the availability of these data, which were used under license for the current study, and so are not publicly available. Data are however available from the authors upon reasonable request and with permission of The British University—faculty of Dentistry.

## Abbreviations

CBCT: Cone beam computed tomography
FOV: Field of view
ICC: Interclass correlation coefficient
